# Recurrent or Obstinate Malarial Attacks

**Published:** 1881-11-20

**Authors:** John H. Pool

**Affiliations:** South Mills, N. C.


### RECURRENT OR OBSTINATE MALARIAL ATTACKS.

BY JOHN H. POOL, M. D., SOUTH MILLS, N. C.

Nothing is more trying to the patience of the physician located in the malarial districts of our Southern country, than the recurrence of "chills and fevers." He is called to a patient suffering from malarial fever, and by the administration of proper remedies the patient is promptly restored. In the course of six or nine or twelve or fifteen days, if the original attack is of the tertian type, the patient appears with a green visage and a shivering frame, and reports a return of the chills. This is repeated until nature is almost worn out or frost puts an end to the trouble (and frost often fails).

Now, what shall we do to prevent our treatment being brought into discredit? By close observation, we will find that tertians recur in six, nine, twelve, fifteen or eighteen days, and in quotidians on the second, fourth, or sixteenth days.

I have been more successful in the treatment of these recurrent chills by the following plan of treatment than from any other: Give fifteen to twenty grains of quinia or cinchonidia three hours before the expected paroxysm. I prefer one large dose to the same quantity given in broken doses at intervals of an hour or two. One large dose will cause less distress to the patient than small doses at short intervals. By giving one large dose we will break up the paroxysm, for none of it will be eliminated by the kidneys until the work is done. But to prevent their return, further treatment is required. On the critical days referred to above, repeat the anti-periodic in ten-grain doses. To reduce the enlarged spleen (which is nearly always present), and to bring the liver into its normal condition, I administer Lugol's solution with ten grains additional of iodide of potassium to the ounce in five or ten drop doses before meals, and three to five dops of Fowler's solution after meals. Without these adjuncts the treatment is apt to prove a failure. On the fourteenth and twenty-first days give the anti-periodic again for a day or two.

For the last five years I have been using largely cinchonidia sulphate, and I have yet to discover that it is not equally as efficient, as a periodic, as quinia sulphate. In the convulsions of children resulting from congestion of the brain in these disorders, I have found nothing better than a combination of chloral hydrate, bromide of potassium and sweet spirits of nitre in appropriate doses.—*Virginia Medical Monthly.*

